# The Effects of Ether on Living Nerves

**Published:** 1887-08

**Authors:** 


### The Effects of Ether on Living Nerves.

Messrs. Vaillard and Pitres are studying neuritis caused by injections of ether in the neighborhood of the nervous trunks of the limbs. These injections have often been followed by stubborn paralysis, and even by trophic disturbance. Experiments made upon guinea pigs show that the anæsthesia and paralysis, which immediately follow the injection, spread rapidly and persist for many weeks or months without either diminution or augmentation. The injection, therefore, determines phenomena identical with those produced by severing the sciatic nerve, of of which we can convince ourselves by a microscopic examination. This shows that the nerve is normal *above* the point where the injection has taken effect. At its point (immediate neighborhood) the lesions vary according to the amount of the substance injected. Some hours after the injection the myelin mingles with the axis cylinder, which is then hardly distinguishable, as are also the nuclei of the inter annular segments. After the fifteenth day the myelin becomes pulvurulent and the nerve sheath is regenerated by special evolution. Below the injection the nerve branches undergo Wallerian degeneration after the sixth day, as in nerve section. To sum up, ether determines necrosis of the fibres which it reaches, and acts upon them as a chemical poison. Perhaps by the side of these grave disadvantages we may acknowledge for these injections a favorable action in frequent cases, as when the elongation of the nerves seems indicated.

This study shows several things of practical importance—first, that the frequently recommended perineural injections of ether may give rise to more grave results than has been supposed; and, second, that in some of the relatively rare cases in which nerve-stretching is indicated, the same result may be attained by the injection of ether.

